# The Role of Maternal Parenting for Children’s Behavior Regulation in Environments of Risk

**DOI:** 10.3389/fpsyg.2020.02159

**Published:** 2020-09-08

**Authors:** Mirjam Deffaa, Mirjam Weis, Gisela Trommsdorff

**Affiliations:** ^1^Developmental and Cross-Cultural Psychology, University of Konstanz, Konstanz, Germany; ^2^Centre for International Student Assessment (ZIB), TUM School of Education, Technical University of Munich, Munich, Germany

**Keywords:** behavior regulation, environmental risk, maternal warmth, restrictive maternal control, mixed-methods

## Abstract

This study investigates the role of maternal parenting and subjective theories for associations between environmental risk and children’s behavior regulation combining a qualitative and quantitative approach. Mothers of 113 primary school children (*M* = 10.06, *SD* = 0.86) in Germany completed questionnaires on parenting, environmental risk, and their child’s behavior regulation. To test for associations, we applied hierarchical regression models. Further, we conducted nine focus groups in settings of high and low environmental risk and used thematic analysis. Maternal warmth showed positive associations with children’s behavior regulation. Restrictive maternal control and children’s behavior regulation were related negatively. The negative association between environmental risk and children’s behavior regulation was partly explained by restrictive maternal control. When maternal warmth was added into the model on environmental risk, restrictive maternal control, and children’s behavior regulation, both maternal parenting practices lost its significant associations with children’s behavior regulation. Qualitative findings gave insights into parents’ subjective theories, suggesting adverse peer effects as possible explanation for the relation between environmental risk and children’s behavior regulation. The results are discussed in terms of their contribution to theoretical considerations on behavior regulation development in different environmental risk settings.

## Introduction

Several favorable developmental outcomes including better academic performance (McClelland and Cameron, 2011), physical health, social status, financial wealth (Moffitt et al., 2013), and social competencies (Blake et al., 2015) have been linked to a high motivation and ability of behavior regulation. Behavior regulation has been defined as the ability and motivation to pay attention, follow rules, resist temptation, and inhibit impulsive behavior to comply with personal goals as well as with environmental demands (Karreman et al., 2006; Weis et al., 2016). Recent findings revealed that children with high environmental risk are more likely to show lower levels of behavior regulation than children with low environmental risk (Størksen et al., 2015; Backer-Grøndahl and Naerde, 2016; Gach et al., 2018). However, underlying mechanisms of the association between environmental risk and children’s behavior regulation remain unknown. The present mixed-method study examines associations between environmental risk, parenting practices (maternal warmth; restrictive maternal control), and children’s behavior regulation. We seek to complement and evaluate quantitative questionnaire data with qualitative focus groups findings to better understand parents’ subjective theories regarding children’s development of behavior regulation and their parenting in different environmental risk settings. Parental subjective theories include implicit beliefs and assumptions about the development of children (Trommsdorff et al., 2012) and fill the developmental niche between sociocultural values and ideas about childhood development and individual parental behavior (Harkness and Super, 2002).

Besides individual characteristics, like a child’s intelligence and temperament, the kind of parenting is of great importance for the development of behavior regulation as it guides the internalization process of autonomous regulation processes (Davidov and Grusec, 2006; Karreman et al., 2006; Lengua et al., 2014; Weis et al., 2016). In fact, early experiences of parent-child dyadic regulation of emotions have been found to function as prototype for later individual self-regulation by fostering the child’s self-efficacy in regulating one’s emotions and behavior (Sroufe, 2005). These early dyadic regulation experiences provide the foundation for a later adaptive development of self-regulation capacities (Causadias et al., 2012; Kiss et al., 2014). In Germany, where we conducted this study, recent social changes have led to a higher involvement of fathers in childcare activities. Nevertheless, mothers remain the main caregivers of children below 18 years in Germany (OECD, 2017). Thus, without neglecting the importance of paternal parenting and involvement for children’s behavior regulation (see e.g., Meuwissen and Carlson, 2015), in the current study we focus on maternal parenting. More specifically, we investigate maternal warmth and restrictive maternal control, that have been found to be associated with children’s behavior regulation. In a longitudinal study by Colman et al. (2006), children who received a higher level of maternal warmth at the age of four, showed higher behavior regulation at the age of 8 years. In contrast, high restrictive maternal control was a predictor for lower behavior regulation in children.

According to the theory of domain-specificity of parenting by Grusec and Davidov (2010), context-specific domains of interaction guide parenting practices as well as children’s behavior. Two important domains for the development of children’s behavior regulation are the domain of control and the domain of reciprocity. The domain of reciprocity is activated in situations in which mother-child interactions seek to accommodate each other’s needs and wishes. Thus, operating in the domain of reciprocity encourages a child’s will to maintain a positive relationship to its mother by showing socially accepted behavior and thereby facilitates the development of behavior regulation (Jennings et al., 2008). Furthermore, experiencing reciprocity fosters self-efficacy, agency, and positive emotions, contributing to the development of behavior regulation (Suchodoletz et al., 2011; Trommsdorff, 2012; Feng et al., 2017). Maternal warmth as parenting practice in the domain of reciprocity can be defined as provision of positive affect and love toward the child (MacDonald, 1992; Suchodoletz et al., 2011; Grusec and Davidov, 2015). The domain of control is activated in situations in which the mother functions as an external source of control (Grusec and Davidov, 2010). Restrictive maternal control, a specific parenting practice used in the domain of control, can be defined as aiming to alter a child’s behavior by high external control, power-assertiveness, and harshness without explanation. Parents who exert high restrictive control do not explain or justify their actions. High restrictive control might inhibit children’s development of autonomous regulation abilities by not granting the opportunity for individual reflection and decision-making to the child (Karreman et al., 2006; Crossley and Buckner, 2012; Marcone et al., 2020). An earlier study showed that the additional use of warm parenting might decrease negative relations between restrictive parenting and children’s behavior regulation (Zubizarreta et al., 2019). In recent studies, maternal emotional regulation capacities have been shown to affect maternal sensitivity to a child’s demands and needs. On the one hand, maternal emotional dysregulation is related to mothers’ own remembered childhood emotion socialization (Tronick, 2007; Riva Crugnola et al., 2019; Leerkes et al., 2020). On the other hand, current contextual factors, such as stressful living conditions might affect maternal parenting capacities, too.

In line with Belsky’s process model of parenting, besides personal developmental history and one’s own personality, parenting is affected by contextual sources of stress that we define as the exposure to environmental risk (Belsky, 2002; Belsky and Jaffee, 2006). Only a few studies on the development of behavior regulation have considered associations between environmental risk and the kind of maternal parenting when investigating children’s behavior regulation (e.g., Lengua et al., 2007; Brophy-Herb et al., 2012; Crossley and Buckner, 2012). As proposed by McLanahan and Percheski’s (2008) family structure model, high environmental risk might increase maternal stress – even leading to mental health issues (e.g., anxiety and depressions; see Rollè et al., 2017) – and thereby might facilitate more ineffective parenting practices like controlling, restrictive, and disapproving parenting practices (Duncan et al., 2015; Harvey et al., 2016). In several studies, mothers in settings with high environmental risk have been found to expect higher levels of obedience and conformity to social expectations from their children than mothers in settings with low environmental risk (Roubinov and Boyce, 2017). However, the relation between environmental risk and maternal warmth remains unclear.

In this study, we define the presence of environmental risk as actual or potential threat of insufficient maternal financial (i.e., income, depts), personal (i.e., individual coping styles in case of demands and stressors), and/or social (i.e., support provided by one’s family or community) resources (Conger et al., 2010). Various maternal sociodemographic characteristics have often been used as a proxy for limited financial, personal, and social resources (Wanless et al., 2011). In Germany, earlier studies have shown relations between the following maternal sociodemographic characteristics and limited resources: having a baby before the age of 21 (Firk et al., 2018); a low level of education [lowest level of German secondary education qualification (“Hauptschulabschluss”) or lower; Minello and Blossfeld, 2017]; having more than three children (Kreyenfeld and Konietzka, 2017). Further, neighborhood disadvantages may lead to a limited availability of social, institutional, and economic resources in the community. Possible indicators of neighborhood disadvantages include a high proportion of single-parent households, a high percentage of children living in households in need of financial aid, a high unemployment rate as well as a high number of young people with migration background as an indication of residential segregation (Vazsonyi et al., 2006; Leventhal et al., 2014; Cuellar et al., 2015; Minh et al., 2017). Limited financial, personal, and social maternal resources might go along with an increased likelihood for their children of failing to reach age-appropriate behavior regulation (Vazsonyi et al., 2006; Conger et al., 2010). Recent research has shown that the cumulation of various risk characteristics measured the possible impact of environmental risk more precisely than the presence of a single risk characteristic (Evans et al., 2013).

Middle childhood has been described as a particularly sensitive phase for environmental risk influences. According to the theory of the *developmental switch* by DelGiudice (2018), an increased sensitivity to the external environment during middle childhood may activate a context-sensitive phenotype of behavior genetics influencing the development of children’s behavior regulation. In fact, research has shown adverse effects of persistent (risk exposure over more than 4 years) as well as concurrent (risk exposure only in the recent 2 years), and intermittent environmental risk (reoccurring risk exposure) on behavior regulation in 10- to 12-year-old children (Ackerman et al., 2004). This is in line with other studies that revealed associations between high levels of environmental risk and low levels of behavior regulation in middle childhood (Størksen et al., 2015; Backer-Grøndahl and Naerde, 2016). Given the assumed importance of the kind of maternal parenting for the development of children’s behavior regulation, the goal of the present study is to contribute to a better understanding of associations between environmental risk and maternal parenting for the development of children’s behavior regulation.

By complementing quantitative questionnaire findings with qualitative focus group narratives, we aim to gain a better understanding of individuals’ parental subjective theories in different environmental risk settings. To investigate subjective parenting beliefs, focus groups offer a unique insight into spontaneous negotiation of socially shared knowledge and norms (Stewart and Shamdasani, 2014). Focus groups have been used in previous studies to exploratively investigate parenting beliefs within a direct social discourse and thereby get insights into narrative and contextualization aspects of parenting (e.g., Parker et al., 2012). However, to our knowledge there exist no studies, which conducted focus groups with the specific focus on parenting regarding children’s behavior regulation so far. In the questionnaire part of this study, we examined the role of maternal parenting for the association between environmental risk and children’s behavior regulation. Firstly, we expected a negative association between environmental risk and maternal warmth (hypothesis 1). Further, we expected that environmental risk was positively associated with restrictive maternal control (hypothesis 2). Moreover, we expected a positive association between maternal warmth and the child’s behavior regulation (hypothesis 3) and a negative relation between restrictive maternal control and the child’s behavior regulation (hypothesis 4). We expected a negative association between environmental risk and children’s behavior regulation (hypothesis 5). Finally, we expected the association between restrictive maternal control and children’s behavior regulation to decrease when maternal warmth was included into the model (hypothesis 6). In addition, we assumed that maternal parenting (i.e., restrictive maternal control and maternal warmth) partly explains the negative association between environmental risk and children’s behavior regulation (hypothesis 7).

In our qualitative focus groups we explored participants’ subjective theories regarding parenting and children’s behavior regulation development to gain a better understanding of the children’s developmental niche (Harkness and Super, 2002). We were especially interested whether participants’ subjective theories about the development of behavior regulation of their children differed between participants in groups with high and low environmental risk (research question 1). Furthermore, we aimed to identify participants’ subjective theories about the effectiveness of parenting practices to foster children’s behavior regulation in groups with high and low environmental risk (research question 2).

## Materials and Methods

### Participants

The questionnaire data collection took place in two German cities. The first data collection was conducted in Konstanz, a small university town in Southern Germany (85,000 inhabitants), where 82 mothers of fourth graders participated. A second data collection took place in Mannheim, a larger industrial town in Southern Germany (300,000 inhabitants). In total, 113 mothers in ten neighborhoods of two cities completed questionnaires on parenting practices, children’s behavior regulation, and sociodemographic characteristics. Mother’s mean age was 41.17 years (*SD* = 5.50). Children’s mean age was 10.06 years (*SD* = 0.86). Concerning the risk factor low level of education, 23 mothers (20.35%) did not complete high school or achieved the lowest level of secondary education (Hauptschulabschluss). The samples in the two cities did not differ significantly with regard to legal status or perceived socioeconomic status or education level.

Focus groups were conducted in the same two German cities. In the small town, two different samples for the questionnaire part and the focus group part were drawn. In the larger city, the participants filled out the questionnaires immediately before the focus groups took place. To compare participants living in settings with high and low environmental risk, focus groups were divided into low risk and high risk groups. Following Guest et al. (2017), we aimed at conducting at least three focus groups for each environmental risk setting (high and low risk groups) as after 3–6 focus groups saturation of content is very likely. In total, 49 parents (46 mothers, 3 fathers) participated in nine focus groups, each including 3–8 persons. In the smaller city, 12 mothers participated in three focus groups. As most of the participants were members of a university’s community network, they were expected to experience low environmental risk (low risk groups). In the larger city, 37 participants (34 mothers, 3 fathers) took part in five focus groups. According to the city’s social map (Stadt Mannheim, 2013), two of the focus groups in the larger city took place in neighborhoods where environmental risk was low and four of them in neighborhoods with high environmental risk. Participants in the high risk groups were significantly younger (*M*_low risk_ = 44.05, *SD*_low risk_ = 3.84; *M*_high risk_ = 39.41, *SD*_high risk_ = 9.84), had a lower education level (total number of school years: *M*_low risk_ = 12.8, *SD*_low risk_ = 1.12; *M*_high risk_ = 9.67, *SD*_high risk_ = 2.67), were more often unemployed (unemployment rate_low risk_ = 4.2%, unemployment rate_high risk_ = 56.0%), and had more children than participants in the low risk groups (*M*_low risk_ = 1.92, *SD*_low risk_ = 0.58; *M*_high risk_ = 3.60, *SD*_high risk_ = 1.63). As only three fathers but 46 mothers participated in the focus groups and only mothers (*N* = 82) were included to the sample of quantitative data collection, the main focus of this study is on maternal parenting.

### Procedure

In the smaller German city, mothers of primary school children received an invitation letter for the questionnaire part of this study through local public-school teachers. In this city, the relevant questionnaire was part of a larger research project on self-regulation development and the data has partly been used in earlier publications on self-regulation development (e.g., Weis et al., 2013,2016). For the focus groups in the smaller city, we contacted parents, who had signed up for the university’s network of developmental research by sending invitations via e-mail. In the larger city, we contacted community social workers and primary school teachers, who distributed the invitation letter to the questionnaire as well as focus group part of this study to mothers of primary school children in their schools or community centers. Mothers in the larger city answered the questionnaires relevant for the present study. Our inclusion criteria for quantitative and qualitative data collection in both cities for participation were having a child between the age of 8 and 12 years and being the main caregiver. Exclusion criteria were defined as the child being diagnosed with a psychological, neuropsychiatric, or learning disorder (reported diagnosis by a health care professional).

Focus groups were conducted by a female moderator and a female observer. Central questions structured the discussion (“What is your understanding of behavior regulation?”; “In which situations and how does your child show behavior regulation?”; “Which parenting practices do you believe are helpful to support a child’s development of behavior regulation?”). The focus groups lasted approximately 1 h. They were audiotaped and afterward transcribed verbatim. Quotations were made anonymous to guarantee confidentiality.

To meet ethical research criteria, methods and the procedure of the present study were reviewed by the ethical committee of the University of Konstanz. Mothers provided written informed consent prior to their participation. All data was treated anonymously. At the end of the whole study, participants were informed in a written letter about the main results.

### Materials

#### Assessment of Behavior Regulation

To measure the child’s behavior regulation the recoded hyperactivity scale of the German version of the Strength and Difficulties Questionnaire (SDQ; Goodman, 1997) by Woerner et al. (2004) was administered. The German version has been shown to be of good validity and reliability in German school populations (Klasen et al., 2013). Mothers were asked to indicate typical behaviors or habits of their child. They rated the five items of the hyperactivity scale on a five-point scale (1 = not at all to 5 = very much), e.g., “My child is restless, overactive, cannot stay still for long.”; “Thinks things out before acting.” The recoded hyperactivity scale of the SDQ has been used to operationalize behavior regulation in earlier studies (e.g., Blake et al., 2015; Weis et al., 2016). For the present study, a Cronbach’s α of 0.79 was determined, which indicates acceptable to good internal consistence (George and Mallery, 1999).

#### Assessment of Parenting

For the assessment of parenting practices we used the Parenting Practice Questionnaire (PPQ) by Robinson et al. (1995). The PPQ describes every-day parenting situations and consists of 11 subscales in total. We asked mothers to indicate their typical parenting practices on a five-point scale (1 = never to 5 = always). For the present study, we included one of the original subscales (“maternal warmth”) as well as the subscale (“restrictive maternal control”) into analyses. The scale “maternal warmth” consists of 11 items (e.g., “I show sympathy when my child is hurt or frustrated.”) and showed an acceptable to good internal consistence of Cronbach’s α 0.79 in the present sample (George and Mallery, 1999). The scale “restrictive maternal control” was developed by Weis et al. (2016) on the basis of the definition by Karreman et al. (2006) to identify harsh and restrictive parenting without explaining parental actions and punishment. It includes eight items of the PPQ by Robinson et al. (1995) like “When my child asks why he/she has to conform, I say: because I said so, or I am your parent and I want you to.” In the present study reliability analyses revealed a Cronbach’s α of 0.76 for this scale, indicating an acceptable to good internal consistence (George and Mallery, 1999).

#### Assessment of Environmental Risk

To measure environmental risk, we asked mothers to provide information on their sociodemographic background. The cut-off values to meet the criteria of high environmental risk in the present study included the following factors: a young age of the mother (younger than 21 years at birth), a low education level, more than three children, and a low perceived socioeconomic status (lower than middle class). Additionally, to account for neighborhood effects, we determined the risk of the neighborhood according to statistical information including the ratio of unemployment, number of young people with migration background, the percentage of single-parent households, and the percentage of children of households in need of financial aid. We defined a neighborhood as high risk neighborhood when at least three of the ratios were above the city’s average (see Supplementary Material). Each participant’s individual environmental risk information for each factor (0 = no risk, 1 = risk) was added and summarized by a single index that ranged from 0 (low environmental risk) to 5 (high environmental risk).

#### Data Analysis

For quantitative data analysis, we first computed Pearson correlations to examine associations between the child’s age, maternal warmth, restrictive maternal control, environmental risk, and the child’s behavior regulation. To test for gender differences (based on the child’s gender) in maternal warmth, restrictive maternal control, environmental risk, and the child’s behavior regulation, we conducted independent *t*-tests. Further, we tested our hypotheses with hierarchical regression models. For all regression models, we checked for the assumption of residual independency, linearity, homoscedasticity as well as lack of multicollinearity as suggested by Field (2013). We found no evidences of assumption violations.

We analyzed focus groups using the qualitative data analysis software MAXQDA as a mix of interpretive induction (e.g., connecting quotes from participants with existent theoretical framework) and abduction (e.g., generating new hypotheses through an open discovery process; Kuczynski and Kerry, 2002). To structure the process, we applied thematic analysis as described by Braun and Clarke (2006) to identify key themes within the data. For qualitative research the trustworthiness of the findings is of special importance (Rolfe, 2006; Elo et al., 2014). In this study, the trustworthiness was enhanced by providing a highly structured coding system as well as a transparent documentation (for example codes for each theme; see Table 1). Moreover, the coding system and data were revised by a second independent rater. The inter-rater reliability was measured using Cohen’s Kappa (κ), ranging between 0.77 and 0.94 for the different focus groups indicating good inter-rater reliability (Bakeman and Gottman, 1997). To integrate qualitative and quantitative methods, we followed the 7th Publication Manual of the American Psychological Association (2020) for mixed-method research.

**TABLE 1:** Frequencies of themes mentioned in the low and high risk focus groups.

Themes and subthemes	Examples	Low risk groups	High risk groups
**1. Development assumptions**			
1.1. Partly predetermined by temperament	“That has a lot to do with a child’s character.” (A3, 201)	100%	100%
1.2. Development through cognitive maturation	“Sometimes it is hard to interfere from the outside, […] it depends a lot on maturation as well.” (B2, 150)	100%	100%
1.3. External influences			
1.3.1. Every-day social experiences	“And then he/she sees ‘I did this wrong,’ so next time he/she will try a different way.” (A4, 69)	100%	100%
1.3.2. Peer interaction			
1.3.2.1. Positive influence of peers	“If siblings are arguing you should keep out of it so that they learn to regulate themselves.” (B3, 69)	100%	25%
1.3.2.2. Negative influence of peers	“The older they get depending of their friends they will […] do it to be cool.” (A3, 143)	20%	100%
**2. Parenting strategies**			
2.1. Provision of daily routines	“Well I believe [.]a certain routine in the daily structure [helps].” (B1, 277)	100%	75%
2.2. Clear rules	“…and then they know if I say it […] the third time I get angry and they get punished, but with an appropriate punishment.” (A2, 57)	100%	100%
2.3. Cognitive strategies			
2.3.1. Encourage perspective taking	“[.] to ask certain questions so that the child can understand its own role in the situation.” (B2, 172)	80%	75%
2.3.2. Encourage perspective taking	“[…] reflecting how you would feel if somebody did this to you.” (A3, 148)	60%	0%
2.4. Cooperative and compromise-seeking parenting	“You always have to make compromises as a parent.” (A2, 150)	60%	0%
Total number of groups		5	4

**N* (participants high risk groups) = 24; *N* (participants low risk groups) = 25. Percentage values indicate the frequency of themes discussed in the groups. A percentage of 100 means that a theme was discussed in 5 of 5 low risk groups or in 4 of 4 high risk groups.*

## Results

### Quantitative Analyses

Descriptive statistics and correlations between environmental risk, restrictive maternal control, maternal warmth, child’s age, and the child’s behavior regulation are shown in Table 2. The child’s age was negatively related to environmental risk (*r* = −0.33, *p* < 0.01) and maternal warmth (*r* = −0.16, *p* < 0.05). Independent *t*-tests revealed no significant gender differences (child’s gender) in maternal warmth (*t*(111) = −1.25; *p* = 0.21), restrictive maternal control (*t*(111) = 1.49, *p* = 0.14), environmental risk (*t*(111) = 0.93, *p* = 0.35), and children’s behavior regulation (*t*(111) = 0.72, *p* = 0.47). For theoretical reasons children’s gender and age were included as control variable into further analyses (e.g., Matthews et al., 2009).

**TABLE 2:** Summary of means, standard deviations, and pearson correlations.

Variable	*M* (*SD*)	1	2	3	4	5
1. Environmental risk	0.85 (0.99)	–				
2. Restrictive maternal control	2.11 (0.61)	0.30**	–			
3. Maternal warmth	4.68 (0.33)	0.06	−0.31**	–		
4. Child’s age	10.06 (0.86)	−0.33**	−0.19*	−0.16*	–	
5. Child’s behavior regulation	2.36 (0.47)	−0.25**	−0.24*	0.16	0.15	–

*N* = 113. *p < 0.05; **p < 0.01.

In two separate regression models, we tested associations between environmental risk and maternal parenting (e.g., maternal warmth and restrictive maternal control). In a first model, we included children’s age (β (standardized coefficient) = −0.17, *p* = 0.10) and children’s gender (β = 0.12, *p* = 0.20) as control variables and environmental risk (β = 0.01, *p* = 0.89) as predictor for maternal warmth in a first single step model (see Table 3). This regression model showed no significant associations (*F*(3,109) = 1.60, *p* = 0.19, adjusted *R*^2^ = 0.02). In the second model, we included children’s age (β = −0.10, *p* = 0.28) and children’s gender (β = −0.12 *p* = 0.20) as control variables and environmental risk (β = 0.25, *p* < 0.01) as predictor for restrictive maternal control (see Table 3). The regression model revealed a positive and significant association between environmental risk and restrictive maternal control (*F*(3,109) = 4.53, *p* < 0.01, adjusted *R*^2^ = 0.09). Further, we calculated single step linear regression models to identify relations between maternal parenting and children’s behavior regulation. First, we entered children’s age (β = 0.18, *p* = 0.06) and children’s gender (β = −0.09, *p* = 0.32) as control variables and maternal warmth (β = 0.20, *p* < 0.05) as predictor for children’s behavior regulation in a single step model (see Table 4). The model showed a non-significant positive relation between maternal warmth and children’s behavior regulation (*F*(3,109) = 2.53, *p* = 0.06, adjusted *R*^2^ = 0.04). In a second single step model with children’s age (β = 0.10, *p* = 0.28) and children’s gender (β = −0.10, *p* = 0.27) as control variables and restrictive maternal control (β = −0.24, *p* = 0.01) as predictor for children’s behavior regulation, we found a significant negative association between restrictive maternal control and children’s behavior regulation (*F*(3,109) = 3.16, *p* < 0.05, adjusted *R*^2^ = 0.06) (see Table 5).

**TABLE 3:** Regression analysis predicting maternal warmth and restrictive maternal control from environmental risk.

	Maternal warmth			Restrictive maternal control		
		
	*B*	*SEB*	β	*B*	*SEB*	β
Constant	5.26	0.40		2.81	0.71	
Child’s age	−0.06	0.04	−0.17	−0.07	0.07	−0.10
Child’s gender	0.08	0.06	0.12	−0.15	0.11	−0.12
Environ-mental risk	0.01	0.03	0.01	0.16	0.06	0.25**

*N* = 113. *B, SE B* = unstandardized coefficients. β = standardized coefficients. Child’s gender: 0 = boy, 1 = girl. **p < 0.01.

**TABLE 4:** Regression analysis predicting children’s behavior regulation from maternal warmth.

	Behavior regulation		
	
	*B*	*SEB*	β
Constant	0.11	0.87	
Child’s age	0.10	0.05	0.18
Child’s gender	−0.09	0.09	−0.09
Maternal warmth	0.28	0.13	0.20*

*N* = 113. *B, SE B* = unstandardized coefficients. β = standardized coefficients. Child’s gender: 0 = boy, 1 = girl. *p < 0.05.

**TABLE 5:** Regression analysis predicting children’s behavior regulation from restrictive maternal control.

	Behavior regulation		
	
	*B*	*SEB*	β
Constant	2.25	0.57	
Child’s age	0.06	0.05	0.10
Child’s gender	−0.10	0.09	−0.10
Restrictive maternal control	−0.18	0.07	−0.24*

*N* = 113. *B, SE B* = unstandardized coefficients. β = standardized coefficients. Child’s gender: 0 = boy, 1 = girl. *p < 0.05.

Furthermore, we tested associations between environmental risk, maternal warmth, restrictive maternal control, and children’s behavior regulation in a hierarchical regression model with three steps. Values for each step as well as explained variance for each step are shown in Table 6. In the first step, we included children’s age (β = 0.07, *p* = 0.48) and children’s gender (β = −0.09, *p* = 0.33) as control variables and environmental risk as predictor variable (β = −0.24, *p* < 0.05; *R*^2^ = 0.08 for step 1), and children’s behavior regulation as dependent variable. In the second step, we included restrictive maternal control (β = −0.19, *p* < 0.05) to predict children’s behavior regulation. In a third step, we entered maternal warmth (β = 0.16, *p* = 0.11). According to our hypotheses, we found a negative association between environmental risk and children’s behavior regulation when controlling for children’s age and gender. When adding restrictive maternal control, that showed a significant negative association with children’s behavior regulation, environmental risk lost its significance. When maternal warmth entered the model, no significant relation between maternal parenting (i.e., restrictive maternal control and maternal warmth) and children’s behavior regulation remained, instead environmental risk showed a significant negative relation with children’s behavior regulation. The overall regression model of children’s behavior regulation with environmental risk, restrictive maternal control, and maternal warmth as predictors reached significance; *F*(5,107) = 3.23, *p* < 0.01. The full model explained 9.00% of the variance of children’s behavior regulation (adjusted *R*^2^ = 0.09).

**TABLE 6:** Regression analysis predicting children’s behavior regulation from restrictive maternal control, maternal warmth, and environmental risk.

	Behavior regulation				
	
	Adjusted*R*^2^	Δ*R*^2^	*B*	*SEB*	β
Step 1	0.05	0.08*			
Constant			2.14	0.55	
Child’s age			0.04	0.05	0.07
Child’s gender			−0.09	0.09	−0.09
Environmental risk			−0.11	0.05	−0.24*
Step 2	0.08	0.03*			
Constant			2.55	0.58	
Child’s age			0.03	0.05	0.05
Child’s gender			−0.11	0.09	−0.11
Environmental risk			−0.09	0.05	−0.19
Restrictive maternal control			−0.15	0.07	−0.19*
Step 3	0.09	0.02			
Constant			1.26	0.99	
Child’s age			0.04	0.05	0.08
Child’s gender			−0.12	0.09	−0.13
Environmental risk			−0.10	0.05	−0.21*
Restrictive maternal control			−0.10	0.08	−0.14
Maternal warmth			0.22	0.14	0.16

*N* = 113. *B, SE B* = unstandardized coefficients. β = standardized coefficients. Child’s gender: 0 = boy, 1 = girl. *p < 0.05.

### Qualitative Analyses

For the qualitative analysis of the focus groups two independent raters developed a coding system that revealed two key themes: Parenting beliefs about the development of behavior regulation and assumptions about parenting practices to enhance behavior regulation. An overview of the themes discussed in the low and high risk focus groups and frequencies is given in Table 1.

Regarding assumptions about the development of behavior regulation, participants in all four high and five low risk groups assumed that behavior regulation was in part determined by a child’s character and developed through cognitive maturation. Moreover, participants in all four high and all five low risk groups emphasized that every-day social experiences would encourage a child to regulate its behavior. A difference between the high and low risk groups was the perceived influence of peers. While participants in all of the five low risk groups believed that social interaction with peers had a positive effect on their child’s behavior regulation, participants in all four high risk groups and only one of the low risk groups reported to worry about negative influences of the child’s peer group on the development of behavior regulation.

The analyses of parental subjective theories concerning the impact of parenting practices on children’s behavior regulation revealed a variety of parental behaviors that participants believed to foster children’s behavior regulation. Parenting practices that were mentioned in all four high and in three of the four low risk groups were the provision of a daily structure and routines that were believed to function as external regulation mechanisms for the child. Clear rules were assumed to be helpful for the development in all five low risk and four high risk groups. Participants in three of the four high and four of the five low risk groups expressed that the reflection of the child on the own behavior and its consequences helped the child to develop a better understanding of its actions and thereby promoted behavior regulation. Parents in three of the five low risk groups stressed the importance of cooperative and negotiating parenting by allowing compromises with the child as well as cognitive stimulation, like the encouragement of perspective taking. These aspects were not discussed in any of the four high risk groups.

## Discussion

### Integration of Results

In this study, we seek to gain new insights on relations between environmental risk, children’s behavior regulation, and the role of maternal parenting within the framework of individuals’ subjective theories and beliefs about childhood development. We mainly defined environmental risk by a low socioeconomic status in the present study. Thus, all the results for environmental risk imply similar results for low socioeconomic status. As hypothesized, we found a significant negative association between environmental risk and behavior regulation with our quantitative data. This finding is in line with other studies conducted in Germany (e.g., German National Academy of Sciences Leopoldina, 2014) and other western countries (e.g., Valiente et al., 2007; Choe et al., 2013; Lengua et al., 2014; Mathis and Bierman, 2015).

As we aimed to gain new insights on possible explanations for the relation between environmental risk and children’s behavior regulation, we took a closer look at maternal self-reported parenting. In a first step, we investigated relations between environmental risk and maternal parenting. Contrary to our hypothesis, we did not find associations between environmental risk and maternal warmth, suggesting that mothers in different environmental risk settings show comparable levels of maternal warmth. This quantitative finding was underlined by our qualitative data as this example from a high risk group shows:

*“Very important [for children’s behavior regulation] is showing affection and listening.”* (Focus group A3, line 145).

Similarly, a longitudinal study by Lengua et al. (2007) on environmental risk, parenting, and effortful control with 2- to 4-year-old children and their mothers found no associations between maternal warmth and environmental risk. As many researchers before us, we hypothesized parenting to differ strongly between high and low risk settings. However, the fact that reported maternal warmth did not show a significant relation to environmental risk might point to higher similarities in maternal parenting across high and low environmental risk settings than are generally expected. In line with this, in our qualitative focus groups participants in high and low environmental risk settings reported similar subjective theories regarding the importance of a variety of parenting strategies for their children’s behavior regulation development.

Interestingly, besides these similarities among participants in high and low risk groups, our quantitative questionnaire data revealed a significant positive association between environmental risk and restrictive maternal control suggesting that the higher the environmental risk, the more restrictive control was reported by mothers. The qualitative data fits with the quantitative finding as the following quote from a high environmental risk focus group demonstrates:

*“And if she does not behave properly, her father hits her.”* (Focus Group A1, line 50).

In line with this result, Hoffman (2014) argued that a high level of control and restriction might be used by parents living in settings with high environmental risk to “[…] protect children from danger and adverse social influences […]” (p. 137). In this study’s qualitative focus groups we found supporting evidence for this line of reasoning. For example, participants of high risk focus groups reported worries concerning the negative influence of peer groups on their children’s ability and motivation to self-regulate their behavior. As one participant in the high risk group described:

*“If your friends say: ‘let’s skip school and do things that are more fun,’ you have to decide for yourself and regulate yourself and say ‘no, I will stay”’* (Focus group A3, line 215).

Hence, the use of restrictive maternal control of mothers living in settings with high environmental risk might be driven by the need of protection rather than by mere power-assertiveness. However, diverging underlying intentions might lead to an impairment of children’s behavior regulation development. According to the theory of domain-specificity of parenting by Grusec and Davidov (2010), conflicts arise when mother and child do not operate in the same interaction domain. This might be the case when the child struggles to regulate its behavior and is in need of reassurance and guidance (protection domain). When the mother, intending to fulfill the child’s need for protection, reacts with parenting practices of restrictive control such practices may be perceived by the child as threatening the child’s autonomy and need for agency (Trommsdorff, 2012) and may lead to a feeling of rejection in the child, inducing reactance and a lower behavior regulation. Thus, the underlying parenting motivation for the use of restrictive maternal control in high risk settings might be driven by a child-oriented motivation of protection. Further, not only child-oriented motivations might be relevant for the use of restrictive maternal control. Likewise, the higher use of restrictive maternal control in high environmental risk settings might be rooted in the lower levels of behavior regulation in children in high environmental risk. This might lead to a more directive and restrictive reaction of maternal parenting causing a higher use of restrictive maternal control. The bidirectionality of maternal parenting and children’s behavior regulation has been pointed out by various studies (e.g., Moilanen et al., 2015; Bechtel-Kuehne et al., 2016). To determine underlying motivational aspects as well as the direction of effects, future studies should investigate possible effects in longitudinal research designs (Maxwell and Cole, 2007).

Regarding relations between maternal parenting and children’s behavior regulation, our quantitative data revealed important information for understanding differences in children’s behavior regulation. Our model for associations between maternal warmth and children’s behavior regulation revealed a non-significant but positive relation. This might be due to the relatively small percentage of children’s behavior regulation variation explained by maternal warmth. However, the regression coefficient of maternal warmth was significantly positive associated with children’s behavior regulation. This is in line with earlier studies (e.g., Davidov and Grusec, 2006; Suchodoletz et al., 2011), and supports the theory by Grusec and Davidov (2010) on the importance of maternal warmth for the development of a child’s motivation to show autonomous behavior regulation according to the mother’s expectations. Furthermore, we found a significant negative association between restrictive maternal control and children’s behavior regulation. Moreover, in our focus groups we found some interesting insights on parents’ subjective theories about the effectiveness of positive parental control for children’s behavior regulation. Specifically, in our low risk groups participants discussed that they believe making compromises and negotiating with the child as equal partner would allow the child to understand and question their decisions and restrictions and thus foster children’s internalization of behavior regulation. This was not discussed in any of the high risk focus groups. Thus, a broader variety and flexibility in parenting strategies might allow parents in the low risk sample to react less restrictive and controlling than parents in our high risk sample. Indeed, parental willingness to engage in compromises with the child has been found to support children’s autonomy and thereby increases the ability and motivation of behavior regulation in children (Grolnick and Raftery-Helmer, 2013). Further, we found a broad accordance of participants in the low risk focus groups on the importance of encouraging perspective taking and self-reflection to strengthen children’s behavior regulation. For instance, a participant in a low risk group stated:

*“[…]I always tell her: How would you feel […] if someone was treating you […] like this? And I think in difficult situations […] this makes it easier for her to handle the situation.”* (Focus group B2, line 185).

In fact, according to the developmental model of self-regulation by Kopp (1982) cognitive aspects such as perspective taking and self-reflection play a crucial role in the process of developing autonomous behavior regulation and may be supported by parenting behavior.

Interestingly, in our quantitative model on associations between environmental risk, maternal parenting, and children’s behavior regulation, the significant negative association between environmental risk and children’s behavior regulation disappeared when restrictive maternal control entered the model. Thus, restrictive maternal control might partly explain relations between environmental risk and children’s behavior regulation. To further investigate this possible mediation effect, longitudinal data is necessary to perform mediation analyses. To our surprise, when entering maternal warmth into the model on associations between environmental risk, restrictive maternal control, and children’s behavior regulation, both maternal warmth and restrictive maternal control showed no significant associations with children’s behavior regulation. Instead, environmental risk and children’s behavior regulation again showed a significant negative association. Possible explanations might be the protective effect of maternal warmth in the context of environmental risk. The protective role of maternal warmth for children’s behavior regulation has been shown in several studies (e.g., Pinquart, 2017). Furthermore, this finding might point to further environmental risk related influences on the development of children’s behavior regulation. For example, the effect of prenatal stress on behavior regulation development has been closely linked to environmental risk factors (Graignic-Philippe et al., 2014; Bridgett et al., 2015). Thus, among a variety of factors, a higher exposure to prenatal stress might contribute to the relation between environmental risk and children’s behavior regulation, as well.

### Strengths and Limitations

Our study contributes to current research by combining quantitative questionnaire data with qualitative data of individuals’ subjective theories about parenting and children’s behavior regulation in different environmental risk settings. This multi-method approach has been highlighted to be of great importance for investigating dyadic and transactional aspects of developmental processes (Yoshikawa et al., 2013). The focus groups with the specific focus on parenting regarding children’s behavior regulation filled a research gap and resulted in new insights.

However, limitations for interpreting the results need to be considered. The information collected for this study depended on mothers’ self-reports and could be biased. Future data collections should include multiple sources of information (e.g., father, teacher, child), observational measures (e.g., experimental measure of behavior regulation) as well as sequential data to investigate domain and sequence specific parenting and children’s behavior regulation. Second, the cross-sectional design of this study as well as the small sample size do not allow to draw generalized or causal conclusions. Future research should investigate characteristics of parenting in different environmental risk settings and its short- as well as long-term effects on the development of children’s behavior regulation. Longitudinal studies with larger, representative samples and multiple sources of information are needed to investigate possible bidirectional associations between parenting and children’s behavior regulation (e.g., Kochanska and Aksan, 1995; Karreman et al., 2006). Further, our findings reveal possible mediation effects for environmental risk, children’s behavior regulation, and maternal parenting that require longitudinal and large sample data in further investigation. Moreover, further influences on the association between environmental risk and behavior regulation such as differences in temperament (Chen and Schmidt, 2015), cognitive abilities (Brito and Noble, 2014), the role and the presence of fathers (Fagan et al., 2011; Meuwissen and Carlson, 2015) as well as specific influences of the child’s peer group (Mason et al., 2014) should be considered in future research. Regarding the combination of our quantitative questionnaire and qualitative focus groups data, we should mention that the classification as high or low risk were similar but not identical in the two research methods. For the quantitative method, we cumulated various environmental risk factors. In the qualitative method, we divided participants into the two groups based on neighborhood statistics. We checked for group differences in environmental risk factors between high and low risk focus groups retrospectively. Nevertheless, the classification between high and low risk groups might not correspond fully.

According to the theory of differential susceptibility, individuals vary in how much they are affected by positive and negative environmental influences. Children as well as mothers with high environmental susceptibility are highly sensitive to negative as well as positive environmental influences (Ellis et al., 2011). Consequently, those children being most affected by adverse environmental risk influences might especially benefit from interventions that enhance a supportive environment (e.g., parenting interventions). Understanding parenting theories and goals as well as parenting practices of both mothers and fathers in settings with high environmental risk can help to design intervention programs to strengthen existing parenting resources and to improve behavior regulation in children at risk.

## Conclusion

This study’s quantitative results show that parenting, especially maternal warmth and restrictive maternal control are important aspects for the development of children’s behavior regulation in different environmental risk setting. Moreover, the qualitative findings lead to interesting new insights and evaluations of the quantitative findings by pointing out to possible explanations. For instance, participants of high risk focus groups reported worries concerning the negative influence of peer groups on their children’s behavior regulation, which could partly explain the higher use of restrictive maternal control of mothers living in settings with high environmental risk. Thus, this study shows that the inclusion of subjective parenting beliefs in developmental psychological studies on behavior regulation can broaden the understanding of the complex and multicausal relations among environmental risk, parenting practices, and children’s behavior regulation.

## Data Availability Statement

The raw data supporting the conclusions of this article will be made available by the authors, without undue reservation.

## Ethics Statement

The studies involving human participants were reviewed and approved by the Ethics Commission at the University of Konstanz. The participants provided their written informed consent to participate in this study.

## Author Contributions

MD: literature review, conceptualization of research question, responsible for data collection of the high risk focus groups, data analysis and interpretation, and preparation of written manuscript. MW: principal investigator of the focus groups, development of study design of focus groups, conceptual input, general supervisory input, and review of manuscript. GT: principal investigator of quantitative research, development of study design and instrument of quantitative research, conceptual input, general supervisory, and review of manuscript. All authors contributed to the article and approved the submitted version.

## Conflict of Interest

The authors declare that the research was conducted in the absence of any commercial or financial relationships that could be construed as a potential conflict of interest.
