# Supplementary material for: The Role of Maternal Parenting for Children’s Behavior Regulation in Environments of Risk
Source: Front Psychol. 2020 Sep 8;11:2159. doi: 10.3389/fpsyg.2020.02159 (PMC7506104; doi:10.3389/fpsyg.2020.02159)
Supplement: Supplementary file 1 [file Data_Sheet_1.docx]

# Supplementary Material

## Table 1

Neighborhood Risk Characteristics for the City Average as well as the Participants’ Neighborhoods of City A

| Risk characteristics | City A (total) | City District A1 | City District A2 | City District A3 | City District A4 | City District A5 |
| --- | --- | --- | --- | --- | --- | --- |
| Ratio of unemployment | 4.2 % | 3.5 % | 2.1 % | *6.0 %* | *7.8 %* | *6.8 %* |
| Ratio young people with migration background | 52.0 % | 43.0 % | 26.0 % | 39.0 % | *67.0 %* | *78.0 %* |
| Ratio of single-parent households | 22.0 % | 21.0 % | 22.5 % | *27.7 %* | *28.3 %* | *27.1 %* |
| Ratio of children of households in need of financial aid | 21.2 % | 4.9 % | 5.8 % | *34.0 %* | *45.9 %* | *35.6 %* |
| **Risk of neighborhood** |  | **Low risk neighbor-hood** | **Low risk neighbor-hood** | **High risk neighbor-hood** | **High risk neighbor-hood** | **High risk neighbor-hood** |

*Note.* Statistical values according to the federal and municipal statistical data in the year 2013 (City A (2013)). Italicized numbers indicate that the ratio is above the city’s average.

## Table 2

Neighborhood Risk Characteristics for the City Average as well as the Participants’ Neighborhoods of City B

| Risk characteristics | City B (total) | City District B1 | City District B2 | City District B3 | City District B4 | City District B5 |
| --- | --- | --- | --- | --- | --- | --- |
| Ratio of unemployment | 2.8 % | 1.6 % | 2.4 % | *2.9 %* | *3.6 %* | *3.9 %* |
| Ratio young people with migration background | 38.0 % | 30.0 % | 36.0 % | *39.0 %* | *52.0 %* | *46.0 %* |
| Ratio of single-parent households | 26 % | 18.7 % | 25.6 % | 23.4 % | *29.2 %* | *29.3 %* |
| Ratio of children of households in need of financial aid | 8.0 % | 2.4 % | 6.0 % | *10.5 %* | *9.4 %* | *8.5 %* |
| **Risk of neighborhood** |  | **Low risk neighbor-hood** | **Low risk neighbor-hood** | **High risk neighbor-hood** | **High risk neighbor-hood** | **High risk neighbor-hood** |

*Note.* Statistical values according to the federal and municipal statistical data in the years 2013-2018 (Bundesagentur für Arbeit (2015); City B (2013); City B (2014); City B (2018)). Italicized numbers indicate that the ratio is above the city’s average.
